# Evaluation of Epidermal Growth Factor Receptor 2 Status in Gastric Cancer by CT-Based Deep Learning Radiomics Nomogram

**DOI:** 10.3389/fonc.2022.905203

**Published:** 2022-07-11

**Authors:** Xiao Guan, Na Lu, Jianping Zhang

**Affiliations:** Department of General Surgery, The Second Affiliated Hospital of Nanjing Medical University, Nanjing Medical University, Nanjing, China

**Keywords:** gastric cancer, HER2 status, deep learning, radiomics, nomogram, computed tomography

## Abstract

**Purpose:**

To explore the role of computed tomography (CT)-based deep learning and radiomics in preoperative evaluation of epidermal growth factor receptor 2 (HER2) status in gastric cancer.

**Materials and methods:**

The clinical data on gastric cancer patients were evaluated retrospectively, and 357 patients were chosen for this study (training cohort: 249; test cohort: 108). The preprocessed enhanced CT arterial phase images were selected for lesion segmentation, radiomics and deep learning feature extraction. We integrated deep learning features and radiomic features (Inte). Four methods were used for feature selection. We constructed models with support vector machine (SVM) or random forest (RF), respectively. The area under the receiver operating characteristics curve (AUC) was used to assess the performance of these models. We also constructed a nomogram including Inte-feature scores and clinical factors.

**Results:**

The radiomics-SVM model showed good classification performance (AUC, training cohort: 0.8069; test cohort: 0.7869). The AUC of the ResNet50-SVM model and the Inte-SVM model in the test cohort were 0.8955 and 0.9055. The nomogram also showed excellent discrimination achieving greater AUC (training cohort, 0.9207; test cohort, 0.9224).

**Conclusion:**

CT-based deep learning radiomics nomogram can accurately and effectively assess the HER2 status in patients with gastric cancer before surgery and it is expected to assist physicians in clinical decision-making and facilitates individualized treatment planning.

## Introduction

Gastric cancer is one of the most common tumors worldwide, ranking fourth in cancer-related deaths (1). Due to the atypical nature of early symptoms of gastric cancer, many patients are already in advanced gastric cancer when they are diagnosed (2, 3). The main treatment for advanced gastric cancer is surgical resection combined with adjuvant chemotherapy or chemoradiotherapy (4). However, despite treatment, the patients with advanced gastric cancer remain poor prognosis (5, 6). HER2 is associated with the poor prognosis in advanced gastric cancer (7). Studies have confirmed that overexpression of HER2 is a significant driver of gastric cancer tumorigenesis (7–9). Trastuzumab combined with standard chemotherapy can significantly improve overall survival in HER2-positive advanced disease (4, 8, 10, 11). The higher the HER2 positivity degree, the greater the treatment effect (8). Thus, the precise identification of HER2 status is critical in the treatment of gastric cancer (12).

Approximately 30% of patients with gastric cancer are HER2-positive (7, 13). In clinical work, immunohistochemistry (IHC) or fluorescence *in situ* hybridization (FISH) are commonly used methods to detect HER2 status, which are invasive and costly (13, 14). Several studies have used positron emission tomography (PET) imaging to try to predict HER2 status, but the results have been inconsistent (15, 16). Therefore, a new noninvasive method is needed to evaluate HER2 status.

Artificial Intelligence is an emerging technology that provides new approaches to oncology research in recent years. Studies have highlighted the importance of identifying imaging biomarkers in oncology (17). CT is widely used in clinical practice and is the routine imaging examination for preoperative evaluation of gastric cancer patients (18). Radiomics can extract features from medical images, showing great potential in oncology practice (19, 20). The limitations of small datasets can be overcome with transfer learning (21). It extracts deep learning features *via* pre-trained convolutional neural networks (CNNs) (22). Research has confirmed that, under certain conditions, the predictive performance of AI models is not inferior to that of human experts (23, 24).

Therefore, this study aimed to develop a CT-based deep learning radiomics nomogram for patients with gastric cancer to preoperatively evaluate the HER2 status. To our knowledge, this has not been reported in any published study.

## Materials and Methods

### Patients

We reviewed the clinical data of the patients with gastric cancer from January 2017 to January 2022 and selected 357 (mean age, 64.18 ± 11.272 years; the range of the ages, 26 – 90 years) patients for this study, including 167 HER2-positive patients (46.78%) and 190 HER2-negative patients (53.22%). Supplementary material detailed the sample size assessment process (**Figure S1**) and the patient inclusion and exclusion criteria. Clinical, pathology, and laboratory data were derived from medical records. The criteria for judging HER2 status were detailed in supplementary material. Three radiologists reviewed the patient’s enhanced CT arterial phase images and reassessed the patient’s T stage and lymph node (LN) status. They all had more than eight years of medical imaging experience. The evaluation processes of the three doctors were independent of each other, and they had no knowledge of the patient’s pathological information. If the opinions of the three doctors were not uniform, we would take the majority opinion as the patient’s final T stage and LN status. Supplementary material detailed the acquisition of CT images.

We obtained informed consent from patients or their relatives and were approved by the Ethics Committee of the Second Affiliated Hospital of Nanjing Medical University (NO. [2022]-KY-009-01). All patient private information was deleted.

### Regions of Interests

**Figure 1:**
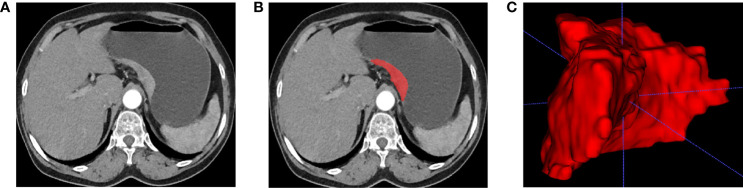
Tumor segmentation. **(A)** Enhanced CT arterial phase images show local gastric wall thickening and enhancement. **(B)** Manual segmentation. **(C)** 3D reconstruction of tumor.

The features extracted from enhanced CT arterial phase images have better predictive performance than portal venous phase (20, 25). Therefore, we resampled the enhanced CT arterial phase images. Two radiologists used ITK-SNAP software to semi-automatically segment the gastric cancer ROI of enhanced CT arterial phase images. Both doctors had more than eight years of medical imaging experience and only knew the location of the pathologically confirmed tumor and had no knowledge of the other information. An example of tumor segmentation was shown in **Figure 1**. Supplementary materials described the details of tumor segmentation.

### Radiomics Features

We used the PyRadiomics package (version 3.0.1) to extract radiomic features from ROIs (26). Most features complied with the Image Biomarker Standardization Initiative (27, 28). We use the pingouin package (version 0.3.11) to calculate the intraclass correlation coefficient (ICC). The features were considered stable with values over 0.8 (**Figure S2**) (29, 30). The process of feature extraction and ICC calculation was described in detail in the supplementary material.

### Deep Learning Features

MobileNetV3, DenseNet201, EfficientNetB7, EfficientNetV2, EfficientNetV2B3, InceptionResNetV2, InceptionV3, NASNet, RegNetX320, RegNetY320, Resnet50, ResNet50V2, VGG16, VGG19 and Xception were used to extract the deep learning features. They were all pre-trained on the ImageNet database only and all convolutional layers were frozen and not trained again (31). The extracted deep learning features were modeled by machine learning methods. Supplementary material described in detail in the pre-training process and feature extraction process of CNN. We visualized the output in a given convolutional layer of CNN by Guided Gradient Weighted Class Activation Mapping (Guided Grad-CAM), which can be explored for important locations in the deep learning function (32).

### Feature Selection and Model Construction

Borderline-SMOTE method was used to deal with the imbalanced data in the training cohort. After normalizing the feature values, we performed feature selection according to the following steps. First, T-test was used for preliminary selecting of features, and features with p<0.05 were selected out. Second, the top 20% of the best features were selected out by univariate analysis. Then we performed recursive feature elimination (RFE) in random forest model and tested using five-fold cross-validation, and these features were evaluated based on accuracy (33). Finally, we performed a 10-fold cross-validation and iterating 100,000 times for “Lambda” parameter tuning in the training cohort to select the optimal “Lambda” parameter of the least absolute shrinkage and selection operator (Lasso) method. We used the Lasso method for feature selection and recorded the feature scores for all patients. We also integrated deep learning features and radiomic features. The specific method was detailed in the supplementary material. The relationship between features and HER2 status was tested with Mann-Whitney U test. SVM or RF was used to construct classification models.

### Nomogram Construction

All clinical, pathological, and laboratory data, including age, sex, tumor location, tumor morphology, albumin, neutrophils, lymphocytes, CEA level, CA724 level, CT-reported LN status, CT-reported T stage and the feature scores were subjected to univariate analysis. The variables with p-values less than 0.05 were assessed by multivariate logistic regression analysis. Then, the nomogram was constructed on this basis.

### Statistical Analysis

Differences between normally distributed variables were assessed by the T-test. Differences in non-normally distributed variables were compared by the Mann-Whitney U test. The differences between categorical variables were assessed by the chi-square test. The feature scores of both cohorts and their probability density distributions were represented by the violin graphs. AUC was used to evaluate the classification models. To validate the stability and generalization of the selected models, a five-fold cross-validation of the entire dataset was performed, and another five-fold cross-validation was performed after shuffling the data. The calibration curve and Hosmer-Lemeshow test were used to evaluate the nomogram. The receiver operator characteristics (ROC) curves were used to evaluate the nomogram, which were assessed by the Delong test. Decision analysis curve (DCA) was drawn to show the value of the classification model and nomogram in clinical application (34).

## Results

### Clinical Characteristics

**Figure 2:**
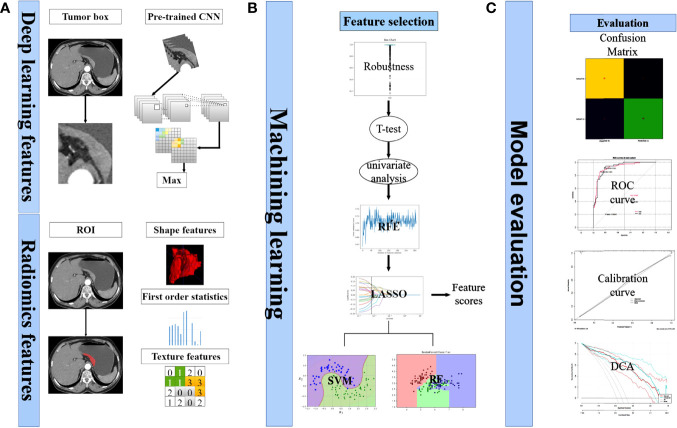
Analysis flowchart. **(A)** Extraction process of deep learning features and radiomics features. **(B)** Selecting of features. Calculation of feature scores. Construction of classification models. **(C)** Model evaluation.

**Figure 2** depicted the workflow of this study. Among these 357 patients, according to the ratio of 7:3, they were assigned into two cohorts at random: training (n = 249) and test (n = 108). **Table 1** summarized the clinical, pathological, and laboratory findings for each cohort. The clinical characteristics of the two groups of patients did not differ significantly.

**Table 1:** Patient characteristics in each cohort.

Clinical characteristics		Train cohort (n = 249)			Test cohort (n = 108)			p
		HER2(+)	HER2(-)	p	HER2(+)	HER2(-)	p	
Age (years), (mean ± SD)		65.25 ± 11.199	63.33 ± 11.243	0.908	65.94 ± 11.323	62.43 ± 11.305	0.776	0.475
Gender, NO (%)				0.924			0.119	0.228
	Male	80 (68.4)	91 (68.9)		41 (82)	40 (69)		
	Female	37 (31.6)	41 (31.1)		9 (18)	18 (31)		
Tumor location, NO (%)				0.528			0.708	0.971
	Fundus	35 (29.9)	41 (31.1)		14 (28)	20 (34.5)		
	Body	28 (23.9)	24 (18.2)		12 (24)	11 (19)		
	Antrum	54 (46.2)	67 (50.8)		24 ()48	27 (46.6)		
Tumor morphology, NO (%)				0.082			0.127	0.950
	Ulceration	99 (84.6)	100 (75.8)		43 (86)	43 (74.1)		
	Flat	18 (15.4)	32 (24.2)		7 (14)	15 (25.9)		
Laboratory tests, median (IQR)								
	Album	40.00 (36.85, 42.70)	40.80 (37.15, 43.80)	0.288	39.35 (35.90, 41.95)	42.50 (37.55, 44.85)	0.013*	0.470
	Neutrophil	3.74 (2.92, 5.10)	3.90 (2.95, 4.81)	0.933	3.92 (2.92, 4.96)	3.59 (2.91, 4.73)	0.707	0.772
	Lymphocyte	1.36 (1.08, 1.78)	1.43 (1.05, 1.85)	0.542	1.23 (0.95, 1.70)	1.47 (1.18, 1.82)	0.036*	0.808
CEA level, NO (%)				0.101			0.339	0.655
	Normal	103 (88)	124 (93.9)		45 (90)	55 (94.8)		
	Abnormal	14 (12)	8 (6.1)		5 (10)	3 (5.2)		
CA724 level, NO (%)				0.000*			0.016*	0.451
	Normal	80 (68.4)	119 (90.2)		37 (74)	53 (91.4)		
	Abnormal	37 (31.6)	13 (9.8)		13 (26)	5 (8.6)		
CT-reported LN status, No (%)				0.576			0.590	0.132
	LN (+)	60 (51.3)	63 (47.7)		19 (38)	25 (43.1)		
	LN (-)	57 (48.7)	69 (52.3)		31 (62)	33 (56.9)		
CT-reported T stage, No (%)				0.000*			0.001*	0.562
	T1-2	41 (35)	96 (72.7)		21 (42)	42 (72.4)		
	T3-4	76 (65)	36 (27.3)		29 (58)	16 (27.6)		
Feature scores median (IQR)		0.11 (-0.02, 0.36)	-0.18 (-0.22, -0.07)	0.000*	1.48 (-0.01, 0.28)	-0.21 (-0.24, -0.16)	0.000*	0.175
								

HER2(+), HER2 positivity; HER2(-), HER2 negativity; LN (+), lymph node metastasis positive; LN (-), lymph node metastasis negative; IQR, interquartile ranges; SD, standard deviation; *p value < 0.05.

### Model Construction and Evaluation

**Figure 3:**
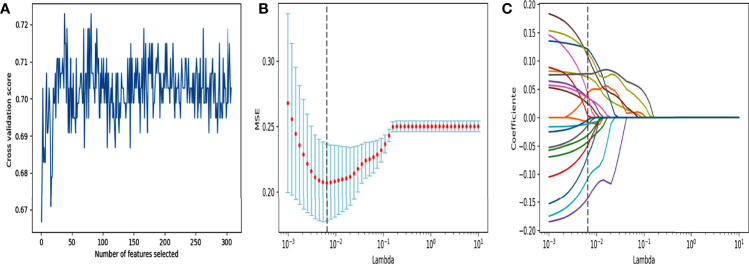
Feature selection using RFE and LASSO. **(A)** RFE feature selection. Select out the features with the highest cross validation scores. **(B)** “Lambda” parameter tuning. MSE: Mean square error. Using 10-fold cross-validation and iterating 100,000 times for “Lambda” parameter tuning of the LASSO method in the training cohort to select the optimal “Lambda” parameter. **(C)** LASSO feature selection. Using the best lambda value for feature selection.

For radiomics model construction in the training cohort, after a series of feature selection (**Figure 3**), a total of 21 features were selected out (**Supplementary Material**). The radiomics-SVM model showed better classification performance in the training cohort, reaching an AUC of 0.8069. Its AUC and accuracy were 0.7869 and 0.8148 in the test cohort (sensitivity, 0.7400; specificity, 0.8793), respectively (**Table S1**).

**Figure 4:**
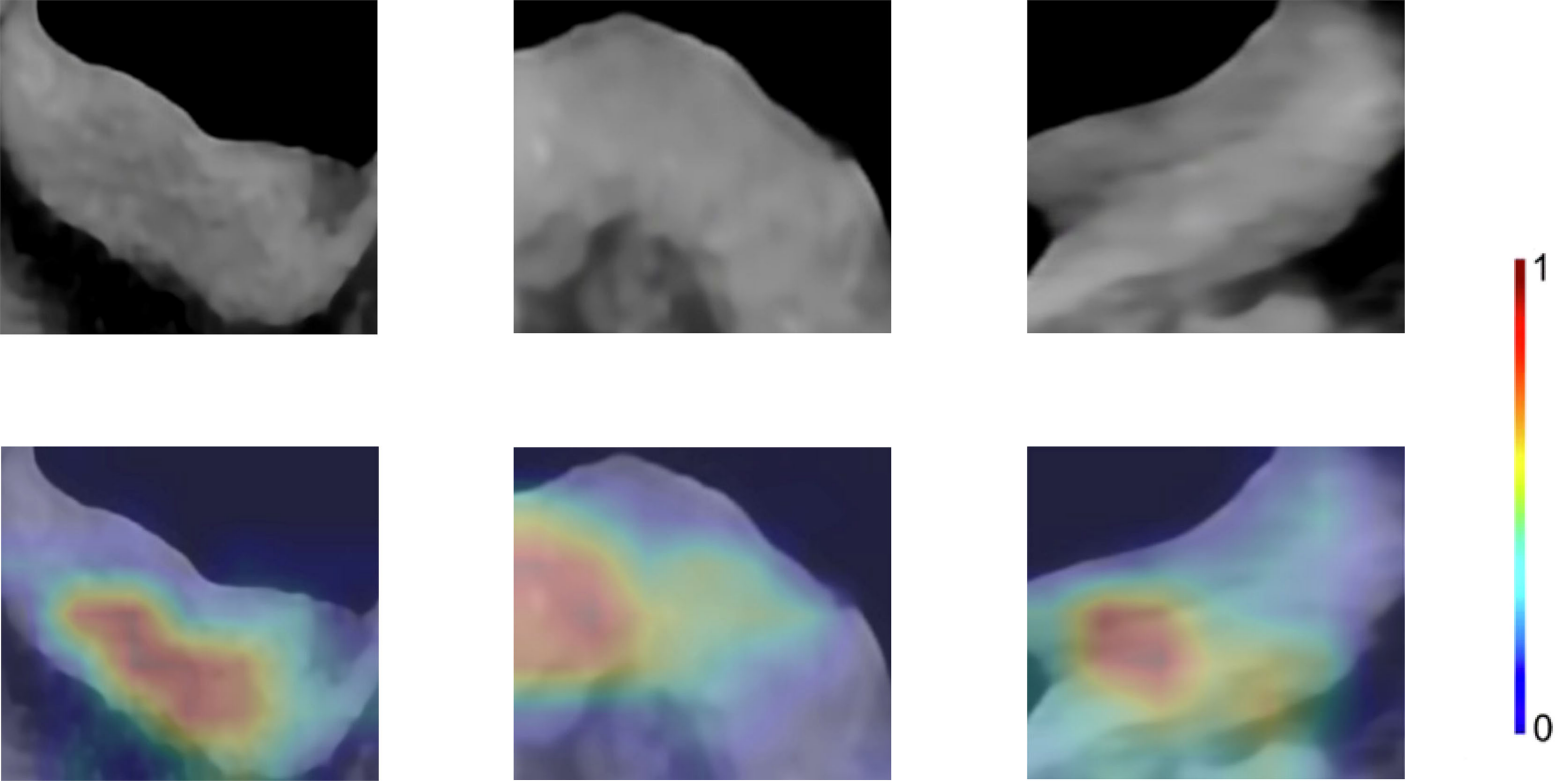
Enhanced CT arterial phase images and feature heatmaps generated from ResNet50. The importance of the feature extracted by the ResNet50 is represented by the color bar.

15 CNNs were used to extract the deep learning feature. The selected features were detailed in the supplementary material. The ResNet50-SVM model had the optimal classification performance (**Table S1**) and outperformed the radiomics model (AUC, 0.8955 vs 0.7869). In the test cohort, its accuracy, sensitivity and specificity were 0.8981, 0.8600, 0.9310, respectively (**Table S1**). For further evaluation of the selected features as well as the selected models, we performed two 5-fold cross-validation on the model. The results show satisfactory stability and accuracy of the radiomics-SVM model and the ResNet50-SVM model (**Table S2**). Feature heatmap indicated the important locations of ResNet50 when generating output (**Figure 4**). Tumors and the surrounding regions were valuable for deep learning feature extraction.

**Figure 5:**
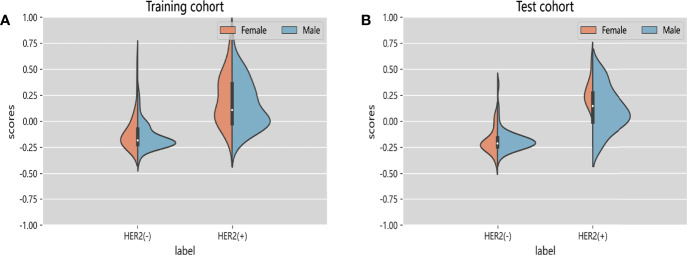
Violin graphs of feature scores of each cohort. **(A)** Training cohort. **(B)** Test cohort. The wider parts of violin graphs indicate that the patients in this group are more likely to adopt the given value and the narrower parts represents the lower probability.

We also tried to integrate radiomics features with deep learning features extracted by ResNet50 to explore whether this method can improve the classification performance. A total of 8 deep learning features and 5 radiomics features were selected for model building (**Supplementary Material**). In test cohort, the Inte-SVM model showed higher classification performance (AUC = 0.9055). Its accuracy also achieved 0.9074 (**Table S1**). We performed two 5-fold cross-validations on the model to evaluate the screened features as well as the Inte-SVM model. The results showed that the Inte-SVM model has great stability and accuracy, and is better than the radiomics-SVM model and the ResNet50-SVM model (**Table S2**). The Inte-feature scores was shown in **Figure 5**. In both cohorts, HER2-positive patients had significantly higher scores than HER2-negative patients. There was a correlation between the features with HER2 status (Mann-Whitney U test, p < 0.001).

### Nomogram Construction and Evaluation

**Figure 6:**
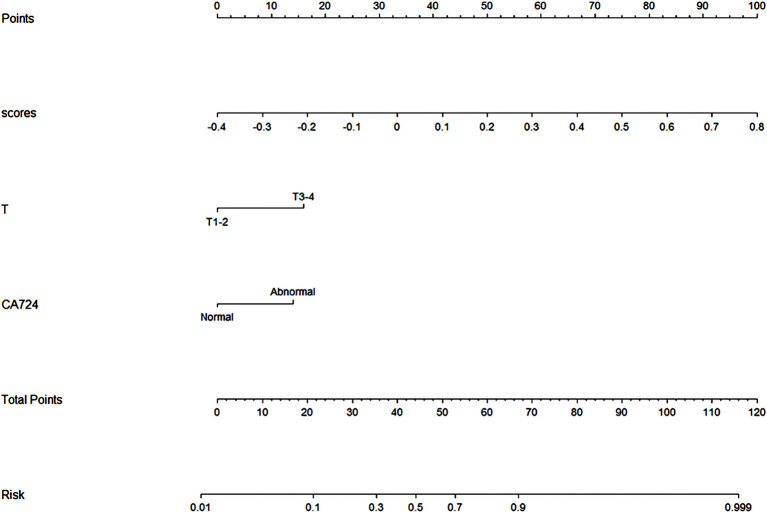
Deep learning radiomics nomogram. Scores: Inte-feature scores. T: CT-reported T stage.

**Figure 7:**
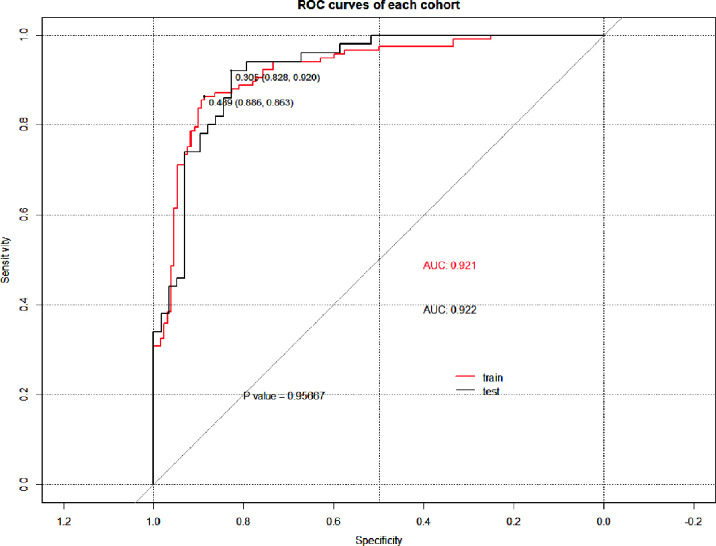
ROC curves of the nomogram in each cohort. p: P value of Delong test.

**Figure 8:**
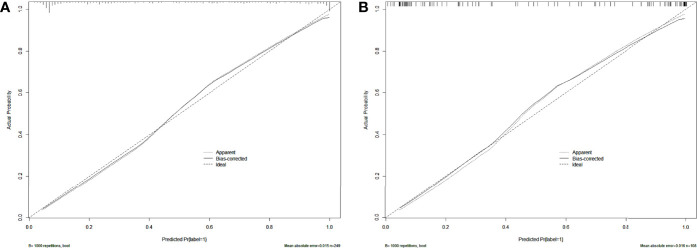
Calibration curves of the nomogram in training **(A)** and test **(B)** cohort. Dashed lines indicate perfect predictions. The prediction performances of the nomogram are represented by solid lines. The solid line and the dashed line are very close, which indicates that the nomogram has excellent predictive performance.

After univariate and multivariate logistic regression analysis, the independent factors were the Inte-feature scores, CT-reported T stage and CA724 level (**Table 2**). The nomogram (**Figure 6**) constructed based on these three independent factors showed excellent classification performance (Hosmer-Lemeshow test, training cohort: p = 0.164; test cohort: p = 0.220). The AUC (**Figure 7**) was higher in both cohort (training cohort: 0.9207; test cohort: 0.9224). The calibration curves indicated that the nomogram had excellent predictive performance (**Figure 8**).

**Table 2:** The results of univariate analysis and multivariate logistic regression analysis.

Characteristics	univariate analysis	logistic regression analysis
	p	p
Age	0.179	–
Gender	0.924	–
Tumor location	0.756	–
Tumor morphology	0.082	–
Album	0.659	–
Neutrophil	0.598	–
Lymphocyte	0.682	–
CEA level	0.102	–
CA724 level	0.000*	0.039*
CT-reported LN status	0.577	–
CT-reported T stage	0.000*	0.004*
Feature scores	0.000*	0.000*

*p value < 0.05.

**Figure 9:**
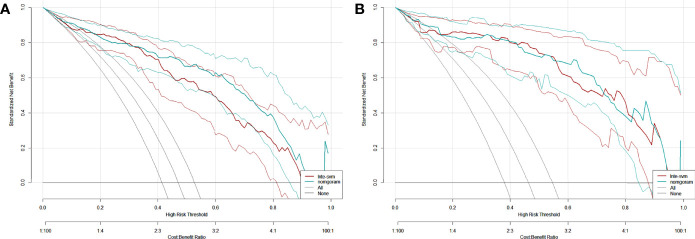
DCA for the Inte-SVM model and the nomogram in training **(A)** and test **(B)** cohort. The gray line indicates that it is assumed that all patients are HER2 positive. The black line indicates that it is assumed that all patients are HER2 negative.

The DCA of the Inte-SVM model and nomogram were shown in the **Figure 9**. The analysis of the results showed that within a certain threshold range, the use of nomogram and Inte-SVM model had a greater net benefit than treat-all or treat-none scheme.

## Discussion

In this study, we developed and verified a CT-based deep learning radiomics nomogram for preoperative evaluation of HER2 status in patients with gastric cancer, which included Inte-feature scores, CT-reported T stage and CA724 level. Deep learning features and radiomic features stratified gastric cancer patients successfully according to their HER2 status. The nomogram facilitated individualized preoperative evaluation of HER2 status.

Accurate and effective HER2 testing plays a crucial role in the treatment and prognosis of patients with gastric cancer (4, 12). Gastroscopic biopsy is a common preoperative test to detect HER2 status. However, it may lead to serious complications such as infection, bleeding, and perforation (35). In recent years, studies have begun exploring the role of PET/CT and magnetic resonance imaging (MRI) in evaluating HER2 status and have achieved certain results (15, 36, 37). However, neither PET/CT nor MRI is a routine preoperative test for patients with gastric cancer. CT is a common method for preoperative evaluation of patients (38). Enhanced CT is more commonly used in tumor therapy (20, 39). Although several research showed that CT-based radiomics can evaluate gene status of lung and colorectal cancers (40, 41), no studies using deep learning and radiomics to evaluate HER2 status in gastric cancer patients had been published to our knowledge.

However, classification using deep learning is difficult to integrate with radiomics. There were two aspects of this problem that must be addressed. First, the most common problem in training CNN models for medical image analysis is the lack of large labeled datasets and training a CNN from scratch on a limited dataset is prone to overfitting (42). Transfer learning is an effective way of solving such problems (21). Deep learning features are extracted through the pre-trained CNNs, and the extracted features are then fed back to supervised machine learning methods like SVM and RF, which greatly reduces the need for large datasets and training time (43). Our study showed that the deep learning features extracted using pre-trained CNNs had good classification performance. At the same time, there is also no study stating which CNN is the most suitable for building classification models. Therefore, we extracted deep learning features by 15 kinds of CNNs. The results confirmed that the ResNet50-SVM was the optimal classification model. As for predicting HER2 status, this model was much better than PET/CT (accuracy, 0.8981 vs 0.644) (15). The AUC of ResNet50-SVM was also higher than that of the MRI-based model (0.8955 vs 0.762) (36) and the conventional enhanced CT (0.8955 vs 0.628) (44). Secondly, we used four methods for feature selecting to avoid model overfitting due to too many features. The analysis of the results confirmed that the classification model constructed using the features selected by the four selecting methods outperformed the model constructed by Wang et al. (45) using a single feature selecting method (AUC, 0.8955 vs 0.830).

Besides, transfer learning can transfer the learned parameters of pre-trained CNN models on a large dataset to solve medical image analysis problems. Among the studies in medical image analysis, migration learning on ImageNet has been the most studied (46). The idea behind transfer learning is that although medical datasets are different from non-medical datasets, the low-level features are universal to most of the image analysis tasks (43). Transferred parameters may serve as a powerful set of features. For the above reasons, we chose Guided Grad-CAM to explore important regions in CT images. The feature heatmap showed that the tumor and its surrounding area were of great value and further research should be carried out based on this result in the future.

Then, the features extracted based on Resnet50 and the radiomics features were integrated to build a new classification model. After integrating the two, the classification performance of the model was improved, which was similar to the studies by Han et al. (47) and Yang et al. (48). Paul et al. (49) suggested that the deep learning features could complement the radiomics features, which are not limited to previously recognized image features and human-understandable attributes. The same view was also expressed by Lao et al. (50). As a result, they will unearth information from medical images that is difficult for humans to notice, increasing the reasonable hope of diagnostic value. But for humans, the meaning of deep learning features is not easy to understand. To this end, we tried to visualize the feature extraction layers of the CNN and drew feature heatmaps to show the important locations in the CNN features (**Figure 4**). In addition to this, radiomic features were also integrated into the model building. Radiomic features are well-defined and pre-selected features that contain predictive information and include image properties that are known or reasonably expected by humans (26). We believed integrating radiomics features and deep learning features was reasonably expected to provide greater value than analyses based solely on radiomics or only deep learning features.

Studies by Huang et al. (51) demonstrated that the integration of multiple markers into one model facilitated individualized management of patients and was superior to the use of a single marker. The study by Liu et al. (52) also came to the same conclusion. The feature scores and clinical factors were subjected to univariate and multivariate logistic regression analysis. The independent factors were the Inte-feature scores, CT-reported T stage and CA724 level. HER2 positivity was associated with higher T stage. The study by Zhang et al. (53) also came to the same conclusion. The aggressive behavior of gastric cancer was associated to HER2 expression, according to the research by Kim et al. (54). The higher the expression of HER2, the stronger the aggressive behavior of gastric cancer and the higher the T stage. Furthermore, the high expression of HER2 correlated with CA724 level, which was consistent with the study by Chen et al. (55).

Due to the low HER2 positive rate in patients with gastric cancer and high price, the detection of HER2 status is not a routine clinical examination (13, 56). Therefore, we combined the Inte-feature scores and the clinical factors to build a nomogram. Both clinicians and patients can use this easy-to-use nomogram, which was in line with the trend of personalized medicine (57). The nomogram had important guiding significance for clinicians to preoperatively evaluate the HER2 status. To evaluate the clinical applicability of Inte-SVM model and nomogram, this study adopted the DCA. Analysis showed that within a certain threshold, compared with treat-all-patients or treat-none scheme, using Inte-SVM model or nomogram to predict HER2 status can provide net benefits.

This study had some notable limitations. First, the deep learning features were obtained by using CNN for transfer learning. Although the test cohort verification proved that the classification model had good predictive performance, there were differences between the source database and the target database. One solution is to build a large database that includes a large number of well-annotated medical imaging data. With the help of this database for CNN training, better performance will be obtained. Then, this was a limited sample size, single-center retrospective study. Further research requires more data and external validation. Besides, this study did not investigate the segmentation performance of the CNN to extract the best layers for feature extraction, nor did it fine-tune the pre-trained CNN using our own CT dataset. Further research on this is required next. Finally, in this study, only enhanced CT arterial phase images were used to extract the deep learning and radiomics features. Other staging of enhanced CT should be studied in the future.

In conclusion, a nomogram was constructed and verified based on deep learning and radiomics feature scores and clinical factors in the study and could assist clinicians to individualize preoperative prediction of HER2 status in gastric cancer patients.

## Data Availability Statement

The raw data supporting the conclusions of this article will be made available by the authors, without undue reservation.

## Ethics Statement

The studies involving human participants were reviewed and approved by The Medical Ethics Committee of the Second Affiliated Hospital of Nanjing Medical University. The Medical Ethics Committee is affiliated to the Second Affiliated Hospital of Nanjing Medical University. The patients/participants provided their written informed consent to participate in this study. Written informed consent was obtained from the individual(s) for the publication of any potentially identifiable images or data included in this article.

## Author Contributions

NL collected and organized the clinical data. XG completed the modeling and data analysis and wrote the manuscript. JZ directed the research. All authors contributed to the article and approved the submitted version.

## Funding

This work was supported by the National Natural Science Foundation of China (NO.81874058).

## Conflict of Interest

The authors declare that the research was conducted in the absence of any commercial or financial relationships that could be construed as a potential conflict of interest.

## Publisher’s Note

All claims expressed in this article are solely those of the authors and do not necessarily represent those of their affiliated organizations, or those of the publisher, the editors and the reviewers. Any product that may be evaluated in this article, or claim that may be made by its manufacturer, is not guaranteed or endorsed by the publisher.
