# Supplementary material for: Evaluation of Epidermal Growth Factor Receptor 2 Status in Gastric Cancer by CT-Based Deep Learning Radiomics Nomogram
Source: Front Oncol. 2022 Jul 11;12:905203. doi: 10.3389/fonc.2022.905203 (PMC9309372; doi:10.3389/fonc.2022.905203)
Supplement: Supplementary file 1 [file DataSheet_1.docx]

Supplementary Material

# Evaluation of sample size

We hypothesized that a deep learning radiomics model could improve the prediction accuracy of HER2 status to 85%, given that the reported accuracy was 64.4% for PET/CT[1]. We performed the proportional power calculation of the binomial distribution. For the training cohort, it had a statistical power of 98.46% and there will be a maximum error of 5%. For the test cohort, it had a statistical power of 80.49% and there will be a maximum error of 5%. For a sample size of all 357 patients, we obtained a statistical power of 99.82%. The sample size for this study was tested to be adequate.

# Inclusion criteria

Enhanced CT examination was performed within one week before gastrectomy, postoperative pathology confirmed gastric cancer, HER2 status was detected by FISH after gastrectomy, no chemotherapy or radiotherapy before operation.

# Exclusion criteria

Poor gastric distension or artefacts in CT images, preoperative radiotherapy or chemotherapy, small gastric cancer lesions that are difficult to identify.

# HER2 status determination

The HER2 status of all gastric cancer patients in this study was tested within one week after gastrectomy using surgical specimens. According to the gastric cancer scoring system, IHC was used to assess HER2 status. An IHC score of 0 or 1+ indicates HER2 negative status, and 3+ indicates HER2 positive status. Cases with an IHC score of 2+ require further FISH testing to confirm HER2 status. If there is gene amplification, it is judged to be HER2 positive, and if there is no gene amplification, it is judged to be HER2 negative.

# CT image acquisition

CT examinations were performed on a 64 Dual Source CT. The patient was instructed to fast for more than 8 hours and to inject anisodamine 20 mg intravenously to avoid gastric motility. Besides, all patients were asked to take 1000ml of warm water orally to dilate the stomach before the examination and hold their breath during the examination. After the non-enhanced abdominal CT scan, the patients were intravenously injected with 1.5 mL/kg of iodinated contrast medium (ioversol injection 320 mg I/mL, Jiangsu Hengrui Pharmaceuticals Co.,Ltd, Jiangsu, China) at a flow rate of 3.0 mL/s by an automatic pump syringe. After the contrast agent injection starts, when the contrast agent concentration reached 100 Hu, the imaging after 8 seconds is the arterial phase, the imaging at 21 seconds after the arterial phase imaging is the venous phase, and the imaging at 90 seconds after the venous phase imaging is the delayed phase. The parameters of the CT scan were as follows: tube voltage 120 kV, tube current 150 - 300 mA, field of view 30 - 50 cm, matrix 512 × 512, rotation time 0.5 seconds, pitch 1.0, and images were reconstructed with section thicknesses of 2 mm.

# Tumor segmentation

Two radiologists with more than 7 years of experience in medical imaging used ITK-SNAP software to delineate the ROI of the tumor. One of the radiologists performed segmentation of lesions in all subjects. Another radiologist delineated tumor ROIs in 30 randomly selected cases from all samples to assess interoperator variability. The entire volume of the tumor was segmented semi-automatically. They drew along the edges of the tumor to obtain the largest cross-sectional area of the tumor. During the delineation process, the air in the stomach, fluid in the stomach, necrotic areas, and adipose tissue in the ROI were excluded. Top and bottom slices were also excluded to reduce bias caused by partial volume effects.

# The categories of image features

**Radiomics features:** First Order Features, Shape Features (2D and 3D), Gray Level Co-occurrence Matrix (GLCM) Features, Gray Level Size Zone Matrix (GLSZM) Features, Gray Level Run Length Matrix (GLRLM) Features, Neighbouring Gray Tone Difference Matrix (NGTDM) Features, Gray Level Dependence Matrix (GLDM) Features. We selected nine filters including Wavelet, Laplacian of Gaussian (LoG) filter, Square, SquareRoot, Logarithm, Exponential, Gradient, LBP2D and LBP3D. The sigma of the LoG filter we chose was 1 and 5.

**Deep learning features:** We removed the last fully connected layer at the top of the network and used global max pooling to take the maximum values of each layer of the feature maps to transform feature maps to raw values. Therefore, the deep learning features did not have specific feature names like the radiomic features, we named them with numbers.

# Feature extraction

Feature extraction includes two types: radiomics feature extraction and deep learning feature extraction. Before performing radiomics feature extraction and deep learning feature extraction, the resampled voxel sizes were set to 1×1×1 mm³ voxels to standardize the slice thickness.

1. Radiomics feature extraction: Radiomics features were calculated from ROIs drawn by radiologists using the open-source python package PyRadiomics. Detailed calculations of handcrafted radiomics features are described and provided in the online documentation of PyRadiomics (https://pyradiomics.readthedocs.io/en/latest/).

We rewrote the “Params.yaml” file provided by the PyRadiomics official website and wrote the types of features that need to be extracted. These features included First Order Features, Shape Features (2D and 3D), Gray Level Co-occurrence Matrix (GLCM) Features, Gray Level Size Zone Matrix (GLSZM) Features, Gray Level Run Length Matrix (GLRLM) Features, Neighbouring Gray Tone Difference Matrix (NGTDM) Features, Gray Level Dependence Matrix (GLDM) Features. We selected nine filters including Wavelet, Laplacian of Gaussian (LoG) filter, Square, SquareRoot, Logarithm, Exponential, Gradient, LBP2D and LBP3D. The LoG filter emphasizes areas of gray level change, where sigma defines how coarse the emphasized texture should be. A low sigma emphasis on fine textures (change over a short distance), where a high sigma value emphasizes coarse textures (gray level change over a large distance). Therefore, we chose 1 and 5.

2. Deep learning feature extraction:

(1) Data preprocessing

After segmenting the tumor area, three consecutive axial slices with the largest tumor area were cropped to 224 mm × 224 mm using a bounding box covering the entire tumor area. 224 mm * 224 mm means the size for the input layer of the CNN models. The cropped images with 3 consecutive axial slices as the image channel were used as the input to the CNN model.

(2) Construction of CNN model

In our study, the 15 CNNs (MobileNetV3, DenseNet201, EfficientNetB7, EfficientNetV2, EfficientNetV2B3, InceptionResNetV2, InceptionV3, NASNet, RegNetX320, RegNetY320, Resnet50, ResNet50V2, VGG16, VGG19 and Xception) mentioned in the manuscript were used for deep learning feature extraction. These networks are pre-trained on the ImageNet dataset, which contains a large number of object categories and manually annotated training images. For broader generalization on other datasets, we did not tune the optimization hyperparameters. After preprocessing, three consecutive slices from CT images with the largest area of tumor lesions are propagated in the network to generate deep learning features.

(3) Acquisition of feature maps

For pre-trained CNN models, the convolutional bases are connected by a fully connected layer. We removed the last fully connected layer. Feature maps were achieved from the new output of this model.

(4) Deep learning feature extraction

By using a global pooling window, CT images are concentrated to a decreased dimensionality. For models with more than one dimensional features, we obtain feature maps with height and width dimensions, which correspond to location invariance in the input layer. After global pooling, each feature map vector is transformed to a maximal raw value among them. The feature maps were transformed to numeric values, which were the representational deep learning features.

# Cropping box

def maskcroppingbox(images_array, use2D=False):

images_array_2 = np.argwhere(images_array)

(zstart, ystart, xstart), (zstop, ystop, xstop) = images_array_2.min(axis=0), images_array_2.max(axis=0) + 1

return (zstart, ystart, xstart), (zstop, ystop, xstop)

# Feature Extraction

def featureextraction(imageFilepath, maskFilepath):

image_array = sitk.GetArrayFromImage(imageFilepath)

mask_array = sitk.GetArrayFromImage(maskFilepath)

(zstart, ystart, xstart), (zstop, ystop, xstop) = maskcroppingbox(mask_array, use2D=False)

roi_images = image_array[zstart - 1:zstop + 1, ystart:ystop, xstart:xstop].transpose((2, 1, 0))

roi_images1 = zoom(roi_images, zoom=[224 / roi_images.shape[0], 224 / roi_images.shape[1], 1], order=3)

roi_images2 = np.array(roi_images1, dtype=np.float)

x = image.img_to_array(roi_images2)

x = np.expand_dims(x, axis=0)

x = preprocess_input(x)

base_model_pool_features = model.predict(x)

feature_map = base_model_pool_features[0]

feature_map = feature_map.transpose((2, 1, 0))

features = np.max(feature_map, -1)

features = np.max(features, -1)

deeplearningfeatures = collections.OrderedDict()

for ind_, f_ in enumerate(features):

deeplearningfeatures[str(ind_)] = f_

return deeplearningfeatures

# ICC analyses

We used the pingouin package to calculate the ICC in the python. The calculation formulas of ICC are as follows:

icc1 = (msb - msw) / (msb + (k - 1) * msw)

icc2 = (msb - mse) / (msb + (k - 1) * mse + k * (msj - mse) / n)

icc3 = (msb - mse) / (msb + (k - 1) * mse)

icc1k = (msb - msw) / msb

icc2k = (msb - mse) / (msb + (msj - mse) / n)

icc3k = (msb - mse) / msb

k: the number of the items. msb, msw, msj, mse were the results of the two-way ANOVA.

# The selected features

**Radiomics features:**

original_glszm_LargeAreaHighGrayLevelEmphasis

original_gldm_SmallDependenceLowGrayLevelEmphasis

wavelet-LHH_firstorder_Uniformity

wavelet-LLH_glszm_LargeAreaHighGrayLevelEmphasis

wavelet-LLH_glszm_SmallAreaLowGrayLevelEmphasis

wavelet-LHL_glcm_Idn

wavelet-LHL_glszm_LargeAreaHighGrayLevelEmphasis

wavelet-HLL_glszm_LargeAreaHighGrayLevelEmphasis

wavelet-HLH_glszm_LargeAreaHighGrayLevelEmphasis

wavelet-HHL_glszm_LargeAreaHighGrayLevelEmphasis

wavelet-LLL_glrlm_ShortRunLowGrayLevelEmphasis

wavelet-LLL_glszm_LargeAreaHighGrayLevelEmphasis

wavelet-LLL_gldm_SmallDependenceLowGrayLevelEmphasis

log-sigma-1-0-mm-3D_glszm_LargeAreaHighGrayLevelEmphasis

exponential_glcm_Idn

exponential_glrlm_RunVariance

exponential_gldm_SmallDependenceLowGrayLevelEmphasis

square_glrlm_RunVariance

square_gldm_LargeDependenceEmphasis

square_gldm_LargeDependenceHighGrayLevelEmphasis

squareroot_gldm_DependenceNonUniformity

**Deep learning features:**

After we had extracted the deep learning features, we named these features from 0 to n-1, with n being the total number of features. For example, if we extracted a total of 512 deep learning features from VGG16, we would name these features from 0 to 511. Due to the poor interpretability of deep learning, there are no specific feature names for deep learning features. We have named them this way for descriptive purposes only, and this naming has no special meaning.

DenseNet201: 196, 344, 464, 734, 1138, 1372, 1618, 1787

EfficientNetB7: 175, 192, 269, 347, 427, 599, 684, 782, 783, 898, 899, 986, 1043, 1107, 1198, 1228, 1244, 1286, 1449, 1656, 1701, 1742, 1801, 1847, 1867, 1910, 1992, 2002, 2011, 2183, 2376, 2543

EfficientNetV2B3: 196, 207, 254, 259, 287, 295, 299, 356, 408, 470, 482, 516, 546, 571, 619, 723, 795, 854, 940, 974, 1050, 1096, 1116, 1200, 1253, 1291, 1339, 1378, 1417, 1523, 1524

EfficientNetV2: 22, 231, 270, 372, 448, 555, 599, 643, 752, 790, 850, 891, 935, 1010, 1059, 1060, 1082, 1227

InceptionResNetV2: 147, 264, 442, 491, 737, 803, 1068, 1192, 1269, 1303, 1314, 1315, 1328, 1388, 1446

InceptionV3: 2, 99, 120, 141, 170, 256, 300, 349, 357, 358, 417, 423, 469, 500, 505, 521, 525, 527, 563, 569, 605, 608, 612, 637, 650, 660, 701, 713, 732, 758, 760, 763, 811, 824, 838, 845, 1046, 1068, 1859, 1893, 1962, 1971, 2000, 2011, 2022, 2024, 2031

MobileNetV3: 24, 75, 176, 320, 526, 706, 1114, 1251

NASNetMobile: 20, 97, 136, 156, 301, 316, 412, 420, 429, 470, 526, 743, 753, 766, 924

RegNetX320: 197, 1342, 1604, 2364, 2366

RegNetY320: 51, 152, 378, 387, 452, 551, 588, 616, 723, 918, 1159, 1217, 1263, 1284, 1350, 1571, 1619, 1646, 1803, 1836, 2186, 2327, 2473, 2777, 2794, 2896, 3110, 3286, 3393, 3425, 3450, 3516, 3588, 3674

ResNet50: 142, 152, 206, 211, 397, 480, 559, 631, 700, 832, 1067, 1329, 1423, 1425, 1610, 1799, 1810, 1900, 1994

ResNet50FC: 38, 95, 116, 248, 477, 498, 558, 977, 1012, 1377, 1635, 1955, 1959

ResNet502C: 12, 63, 69, 137, 145, 155, 216, 224, 231, 244

ResNet503D: 116, 180, 261, 381

ResNet504F: 68, 134, 302, 342, 351, 382, 387, 415, 669, 677, 792, 922, 1000

ResNet50V2: 15, 37, 41, 86, 116, 170, 182, 259, 286, 341, 350, 408, 516, 538, 579, 585, 702, 761, 826, 847, 896, 908, 921, 1082, 1268, 1342, 1364, 1467, 1546, 1659, 1709, 1819, 1906, 1907, 1989

VGG16: 226, 250, 260, 307, 309, 368

VGG19: 7, 23, 65, 88, 155, 333, 414, 445, 450, 477, 497

Xception: 38, 95, 116, 248, 498, 558, 977, 1012, 1955, 1992

**Integration of features:**

wavelet-LHL_glcm_Idn

wavelet-HLL_glszm_LargeAreaHighGrayLevelEmphasis

exponential_glrlm_RunVariance

exponential_gldm_SmallDependenceLowGrayLevelEmphasis

square_gldm_LargeDependenceEmphasis

ResNet50: 211, 559, 631, 1329, 1423, 1425, 1799,1900

# Feature scores

For convenience, we will use D to represent ResNet50.

Scores = wavelet-LHL_glcm_Idn*0.060448143 – wavelet-HLL_glszm_LargeAreaHighGrayLevelEmphasis*0.029397596 + exponential_glrlm_RunVariance*0.059329577 + exponential_glrlm_RunVariance*0.05082447 + square_gldm_LargeDependenceEmphasis*0.273149471+ D211*0.063157361 + D559*0.013416019 + D631*0.023803517 + D1329*0.000103967 + D1423*0.01768579 + D1425*0.018199239 + D1799*0.034958938 + D1900*0.004524292

# Supplementary Table S1

Table S1: The predictive performance of models in test cohort

|  | AUC | Accuracy | Sensitivity | Specificity | PPV | NPV | F1-score |
| --- | --- | --- | --- | --- | --- | --- | --- |
| radiomics-SVM | 0.7869 | 0.8148 | 0.74 | 0.8793 | 0.8409 | 0.7969 | 0.7872 |
| radiomics-RF | 0.771 | 0.7778 | 0.68 | 0.8621 | 0.8095 | 0.7576 | 0.7391 |
| DenseNet201-SVM | 0.8569 | 0.8611 | 0.8 | 0.9138 | 0.8889 | 0.8413 | 0.8421 |
| DenseNet201-RF | 0.8669 | 0.8704 | 0.82 | 0.9138 | 0.8913 | 0.8548 | 0.8542 |
| EfficientNetB7-SVM | 0.8669 | 0.8704 | 0.82 | 0.9138 | 0.8913 | 0.8548 | 0.8542 |
| EfficientNetB7-RF | 0.8196 | 0.8241 | 0.76 | 0.8793 | 0.8444 | 0.8095 | 0.8 |
| EfficientNetV2B3-SVM | 0.8855 | 0.8889 | 0.84 | 0.931 | 0.913 | 0.871 | 0.875 |
| EfficientNetV2B3-RF | 0.8669 | 0.8704 | 0.82 | 0.9138 | 0.8913 | 0.8548 | 0.8542 |
| EfficientNetV2L-SVM | 0.8096 | 0.8148 | 0.74 | 0.8793 | 0.8409 | 0.7969 | 0.7872 |
| EfficientNetV2L-RF | 0.7724 | 0.7778 | 0.7 | 0.8448 | 0.7954 | 0.7656 | 0.7447 |
| InceptionResNetV2-SVM | 0.7769 | 0.8148 | 0.72 | 0.8966 | 0.8571 | 0.7879 | 0.7826 |
| InceptionResNetV2-RF | 0.7883 | 0.7963 | 0.68 | 0.8966 | 0.85 | 0.7647 | 0.7556 |
| InceptionV3-SVM | 0.8869 | 0.8796 | 0.84 | 0.9138 | 0.8936 | 0.8688 | 0.866 |
| InceptionV3-RF | 0.8696 | 0.8704 | 0.86 | 0.8793 | 0.86 | 0.8793 | 0.86 |
| MobileNetV3Large-SVM | 0.8179 | 0.8056 | 0.82 | 0.7931 | 0.7736 | 0.8364 | 0.7961 |
| MobileNetV3Large-RF | 0.8324 | 0.8333 | 0.82 | 0.8448 | 0.82 | 0.8448 | 0.82 |
| NASNetMobile-SVM | 0.8396 | 0.8333 | 0.82 | 0.8448 | 0.82 | 0.8448 | 0.82 |
| NASNetMobile-RF | 0.821 | 0.8241 | 0.78 | 0.8621 | 0.8298 | 0.8197 | 0.8041 |
| RegNetX320-SVM | 0.8583 | 0.8611 | 0.82 | 0.8966 | 0.8723 | 0.8524 | 0.8454 |
| RegNetX320-RF | 0.8683 | 0.8704 | 0.84 | 0.8966 | 0.875 | 0.8667 | 0.8571 |
| RegNetY320-SVM | 0.8669 | 0.8704 | 0.82 | 0.9138 | 0.8913 | 0.8548 | 0.8542 |
| RegNetY320-RF | 0.8455 | 0.8518 | 0.76 | 0.931 | 0.9048 | 0.8182 | 0.8261 |
| ResNet50-SVM | 0.8955 | 0.8981 | 0.86 | 0.931 | 0.9149 | 0.8852 | 0.8866 |
| ResNet50-RF | 0.8855 | 0.8889 | 0.84 | 0.931 | 0.913 | 0.871 | 0.875 |
| ResNet50V2-SVM | 0.7983 | 0.8241 | 0.74 | 0.9138 | 0.8604 | 0.8 | 0.7957 |
| ResNet50V2-RF | 0.8196 | 0.8241 | 0.76 | 0.8793 | 0.8444 | 0.8095 | 0.8 |
| VGG16-SVM | 0.8569 | 0.8611 | 0.8 | 0.9138 | 0.8889 | 0.8143 | 0.8421 |
| VGG16-RF | 0.8296 | 0.8333 | 0.78 | 0.8793 | 0.8478 | 0.8226 | 0.8125 |
| VGG19-SVM | 0.8655 | 0.8704 | 0.8 | 0.931 | 0.9091 | 0.8438 | 0.8511 |
| VGG19-RF | 0.8569 | 0.8611 | 0.8 | 0.9138 | 0.8889 | 0.8413 | 0.8421 |
| Xception-SVM | 0.8783 | 0.8796 | 0.86 | 0.8966 | 0.8776 | 0.8814 | 0.8687 |
| Xception-RF | 0.8768 | 0.8796 | 0.84 | 0.9138 | 0.8936 | 0.8688 | 0.866 |
| Inte-SVM | 0.9055 | 0.9074 | 0.88 | 0.931 | 0.9167 | 0.9 | 0.898 |
| Inte-RF | 0.8968 | 0.8981 | 0.86 | 0.931 | 0.9149 | 0.8852 | 0.8866 |

Inte: the model built with the integrated features. PPV: positive predictive value; NPV: negative predictive value.

# Supplementary Table S2

TableS2: The AUC of the models in two 5-fold cross-validation

| Radiomics-SVM | ResNet50-SVM | Inte-SVM |
| --- | --- | --- |
| 5-fold cross-validation | | |
| 0.7830 | 0.8969 | 0.9069 |
| 0.7854 | 0.8955 | 0.9074 |
| 0.7909 | 0.8981 | 0.9166 |
| 0.8060 | 0.9056 | 0.8980 |
| 0.7603 | 0.9000 | 0.9000 |
| 5-fold cross-validation after shuffling the patients | | |
| 0.7878 | 0.8756 | 0.9262 |
| 0.7969 | 0.8990 | 0.9055 |
| 0.7752 | 0.8862 | 0.9150 |
| 0.8094 | 0.8937 | 0.8980 |
| 0.8058 | 0.8819 | 0.9006 |

## Supplementary Figure S1

##
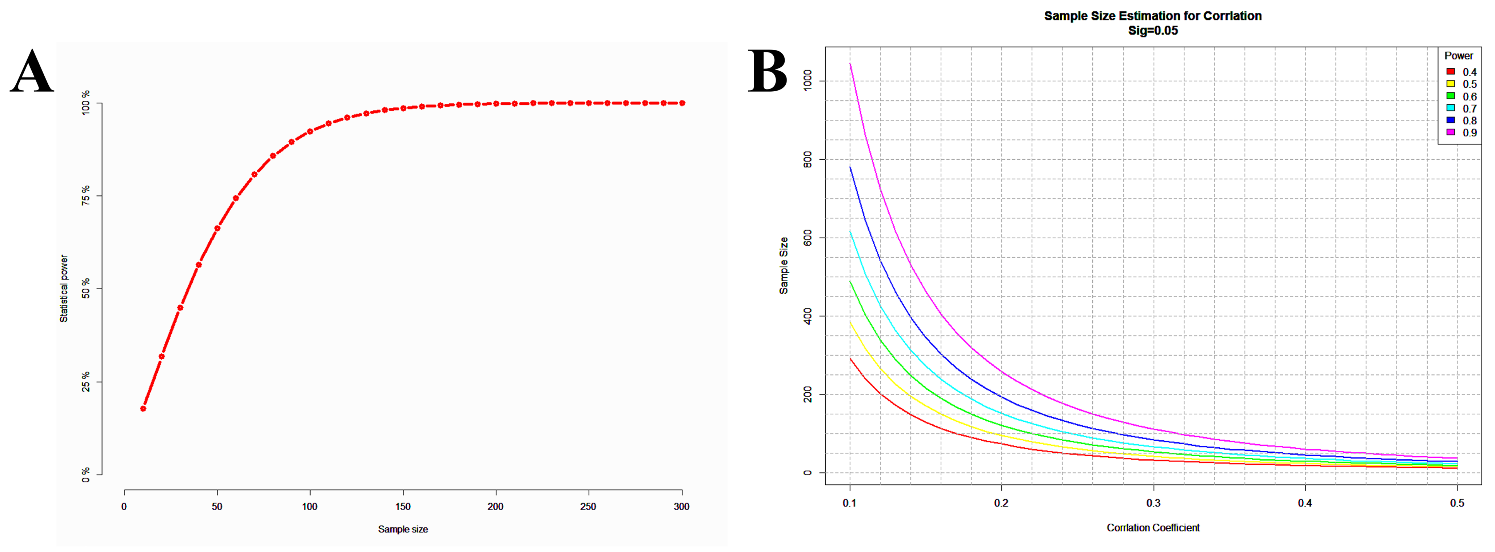


**Supplementary Figure 1.** Data visualization between statistical power and sample size. (A) When the sample size reaches 200, with the increase of the sample size, the statistical power of this study does not change significantly. (B) At 40% statistical power, about 75 samples are required to detect a correlation of 0.2, and at 90% statistical power, about 260 samples are required to detect the same correlation.

## Supplementary Figure S2

##
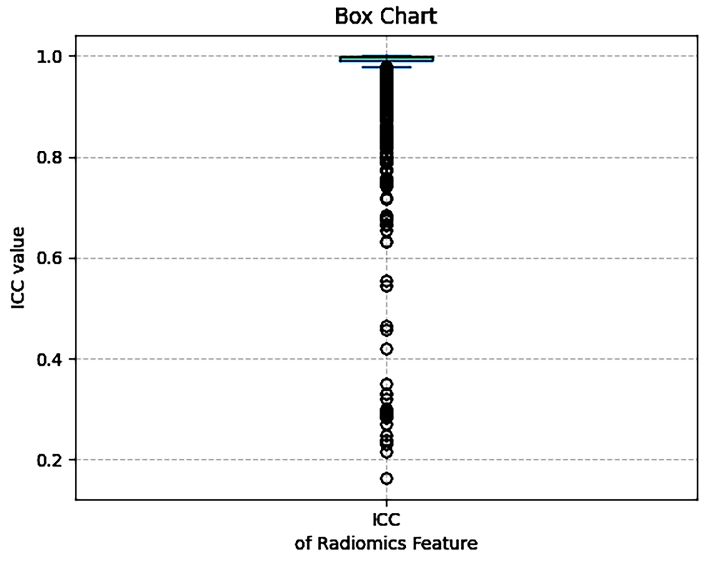


**Supplementary Figure 2.** Distribution of ICC values for radiomic features.

# Reference

1. Chen, R.; Zhou, X.; Liu, J.; Huang, G., Relationship Between 18F-FDG PET/CT Findings and HER2 Expression in Gastric Cancer. *Journal of nuclear medicine : official publication, Society of Nuclear Medicine* **2016,** *57* (7), 1040-4. doi: 10.2967/jnumed.115.171165
